# Amyloid Beta: Multiple Mechanisms of Toxicity and Only Some Protective Effects?

**DOI:** 10.1155/2014/795375

**Published:** 2014-02-05

**Authors:** Paul Carrillo-Mora, Rogelio Luna, Laura Colín-Barenque

**Affiliations:** ^1^Departamento de Neurorrehabilitación, Servicio de Rehabilitación Neurológica, Instituto Nacional de Rehabilitación, 14389 México, DF, Mexico; ^2^Departamento de Neurociencia Cognitiva, Instituto de Fisiología Celular-Neurociencias, Universidad Nacional Autónoma de México, 04510 México, DF, Mexico; ^3^Departamento de Neurociencias, FES Iztacala, Universidad Nacional Autónoma de México, 54090 Tlalnepantla, MEX, Mexico

## Abstract

Amyloid beta (A**β**) is a peptide of 39–43 amino acids found in large amounts and forming deposits in the brain tissue of patients with Alzheimer's disease (AD). For this reason, it has been implicated in the pathophysiology of damage observed in this type of dementia. However, the role of A**β** in the pathophysiology of AD is not yet precisely understood. A**β** has been experimentally shown to have a wide range of toxic mechanisms *in vivo* and *in vitro*, such as excitotoxicity, mitochondrial alterations, synaptic dysfunction, altered calcium homeostasis, oxidative stress, and so forth. In contrast, A**β** has also shown some interesting neuroprotective and physiological properties under certain experimental conditions, suggesting that both physiological and pathological roles of A**β** may depend on several factors. In this paper, we reviewed both toxic and protective mechanisms of A**β** to further explore what their potential roles could be in the pathophysiology of AD. The complete understanding of such apparently opposed effects will also be an important guide for the therapeutic efforts coming in the future.

## 1. Introduction

Alzheimer's disease (AD) is the first cause of cognitive impairment worldwide. Advanced age is still considered the most important risk factor for the disease [1]. In the future, the increase in the lifespan expectancy would therefore raise the number of persons at risk of developing the disease. Thus, it is estimated that the quantity of patients with AD will increase day after day throughout the following decades [2]. For this reason, both scientific and clinical research covering all aspects related to AD has become highly relevant and they have also expanded tremendously in the last decades. So far, the etiology of AD remains unknown. However, many factors have been involved and also some hypotheses have been proposed trying to explain the beginning and progression of the neurodegenerative process observed in this disorder [3]. One of these hypotheses is the “amyloid hypothesis,” which supports the idea that beta-amyloid peptide (A*β*) plays a very important role in the origin and progression of the nervous tissue damage seen in these patients [4]. Some evidences support this hypothesis: (1) the demonstration of A*β* as the principal component of both the neuritic plaques and the amyloid angiopathy observed in the AD patients [5]; (2) the observations in which mutations responsible for familial forms of AD drive in some way an increase in the A*β* production [6]; and (3) the several toxic effects that A*β* has shown both at *in vitro* as *in vivo* experiments, which reproduce some of the observed alterations in AD [7]. Such evidences suggest that either an excessive production of A*β* or impairment in its adequate clearance could be the key events in the origin and progression of the neuronal damage. However, in a parallel way, some other experimental studies showed that under certain conditions A*β* may instead have positive and even neuroprotective effects in the neural physiology [7]. Moreover, clinical experience based on antiamyloidogenic therapies so far tested has shown only a modest benefit over cognitive impairment or disease's progression, even though some of them have significantly reduced the brain levels of A*β*. Unexpectedly, some of these therapies may instead accelerate cognitive impairment. Despite previous facts and due to the concomitant presence of important side effects during the clinical trials, it is still difficult to categorically conclude that the antiamyloidogenic strategies have failed either because of a lack of efficacy by the side-effect profile or both [8]. Although all these scientific results seem contradictory, it is evident that A*β* has an important role in the pathophysiology of AD. Nevertheless, A*β*'s precise physiology and pathology, as well as its potential intervention in the origin and damage progress of AD are still unknown. Hence, in the current paper we pretend to review the mechanisms of toxicity and protection that A*β* has demonstrated experimentally in an effort to remark on the elements that may potentially underlie this dual behavior.

### 1.1. Alzheimer's Disease

AD is the main cause of dementia worldwide; it represents 75–80% of the total cases of dementia, affects 5% of the population older than 65 years, and even 30% of the population older than 85 years [2]. The disease incidence has also increased in the last decades due to the higher lifespan expectancy, among other grounds. Moreover, it is that estimated this incidence would increase approximately every 20 years [9]. Currently, world prevalence of AD is calculated to be higher than 24.3 million patients, with an annual incidence of 4.6 million new cases [1]. By 2001, more than 60% of AD cases were found in developing countries and, according to some predictions, such a number will augment until 71% by the year 2040. Total costs expended in health assistance services for AD patients are estimated to be between 5.6 and 88 billions dollars per year, with a per-patient cost fluctuating in between 1,500 and 91,000 dollars per year [10].

It is estimated that 90% of AD cases are sporadic and only 10% exhibit some inherited pattern (usually autosomal dominant type) and is also commonly linked with an early onset (<65 years) [11]. Most of the AD cases have a late onset (94%, approximately), and by far, only the *ε*4-allele polymorphism of the apolipoprotein E (APOE4) has been consistently associated with an increment in the risk for developing the sporadic form of AD; yet recently some other chromosomal *loci* associated with the disease (chromosomes 1, 7 and 8) have been described [12]. Despite the fact that Mendelian inheritance patterns can be seen (more often autosomal dominant), the late onset of AD tends to be considered as a polygenic and multifactorial disease [13]. It is estimated that mutations of the amyloid precursor protein (APP) and the presenilins 1 (PS1) and 2 (PS2), located at chromosomes 21, 14, and 1, respectively, are responsible for up to 71% of early-onset AD cases; however, they could only explain 0.5% for AD total cases.

Even though the physiopathogenic mechanisms responsible for AD onset are still not known in detail, a great variety of possible implicated factors are currently discussed: (a) genetic (mutations and alleles); (b) abnormal deposit of proteins and peptides, which may have toxic effects (A*β*, phosphorylated tau protein, etc.); (c) exogenous toxic elements (aluminum and mercury); (d) oxidative stress (antioxidant deficiency, transition metals, mitochondrial disorders, etc.); (e) vascular disorders (ischemia, hypertension, hyperhomocysteinemia, etc.); (f) trophic factors deficiency; (g) infectious-inflammatory processes (cytokines, virus, etc.), and (h) metabolic disorders (diet, dyslipidemia, diabetes, etcetera) [3]. From the histopathological point of view, the cerebral changes that characterize AD are (1) presence of A*β* peptide of 38–43 amino acids deposits (amyloid plaques, either neuritic or diffuses); (2) presence of intracellular neurofibrillary tangles, which are abnormal deposits of helical filaments of microtubule-associated protein, so-called *tau *protein, which is abnormally hyperphosphorylated and whose normal function is to stabilize the microtubules; (3) amyloid angiopathy, and (4) neuronal granulovacuolar degeneration with Hirano's bodies, among others [14]. Such pathologic changes have a topographic distribution and temporal evolution that are characteristic of the AD; nevertheless, depending on the pathological aspect being studied (amyloid plaques, neurofibrillary tangles, etc.), this distribution can vary widely. In general, it can be said that all these changes are mainly located at the transentorhinal cortex, the hippocampus, the amygdalae, the anterior basal brain and, ultimately, even at the diencephalic nuclei, the brainstem, and the striatum nuclei [14, 15].

### 1.2. Amyloid Hypothesis

**Figure 1:**
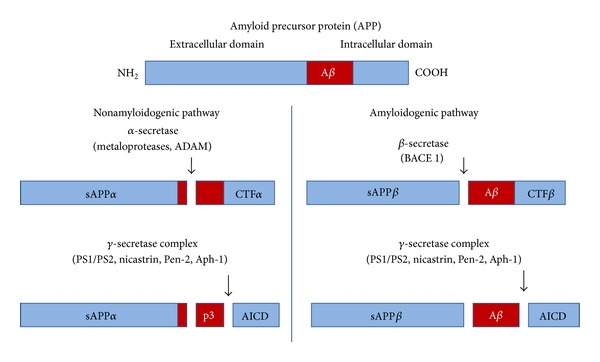
Schematic diagram showing the two proteolytic pathways of amyloid precursor protein: amyloidogenic and nonamyloidogenic.

The history of this hypothesis began with the isolation and identification of a protein-like material that was deposited in the AD patients' meningeal vessels [16]. It was later demonstrated that this material was identical to that obtained from blood vessels of Down syndrome patients, a disorder that is not only characterized by cognitive impairment but is also associated with a trisomy of chromosome 21 [17]. Subsequently, other studies confirmed that this was the same peptide found in senile plaques of AD patients [18, 19]. Finally, the identification of both the protein precursor from which A*β* is originated (the APP) [20, 21] and the first mutation that was associated with AD development (located in the APP gene, precisely), inevitably led to suggest that this peptide has a central role in the disease origin [22]. The amyloid hypothesis was proposed formally for the first time by Hardy and Allsop in 1991, and it still continues to be one of the etiologic hypotheses best scientifically supported nowadays. This hypothesis states that production and excessive accumulation of A*β*, both intracellular and extracellular, as well as under different physical and aggregation states, are some of the beginning events that drive the progressive neuronal damage which fully characterizes the disease [4, 23, 24]. The A*β* is a peptide of 39 to 42 amino acids and is usually produced in all neurons through sequential proteolytic processing of a membrane-attached type-1 protein, called amyloid precursor protein (APP), by means of two enzymatic complexes: the *β* and *γ* secretases [6]. The APP can be processed through two enzymatic pathways, the non-amyloidogenic pathway and the amyloidogenic pathway (Figure 1). Within the non-amyloidonegic pathway, the first step of the proteolysis is mainly performed by enzymes holding *α*-secretase activity (primordially ADAM 10). These enzymes cut the APP within the ectodomain, which correspond to the A*β* fragment. This process produces bigger soluble fragments, thus avoiding the formation of smaller fragments like the A*β* [25]. The *α*-secretase's action releases the extracellular N-terminal domain of the APP, so-called soluble s*α*APP, which possesses different neurotrophic and neuroprotective properties. In addition, the C-terminal fragment of APP that remains anchored to the membrane (C83 o CTF*α*) is once again proteolyzed by the *γ*-secretase producing the fragments p3 (A*β* 17–40/42), which have low-potency cellular toxic properties. Simultaneously, the intracellular domain of the APP (AICD), which has demonstrated some neuroprotective properties, is released inside the cell [25] (Figure 1). In the so-called amyloidogenic pathway, the APP is first proteolyzed by the *β*-secretase (also known as aspartyl protease BACE1), which generates a soluble fragment from the N-terminal domain called sAPP*β* as well as the CTF*β* fragment that remains attached to the membrane. The latter is next proteolyzed by the *γ*-secretase complex then produces the A*β* [26]. The *γ*-secretase is composed of a four proteins complex: Nicastrin, PEN-2, Aph-1, PS1, and PS2, from which both presenilins represent the catalytic site of the enzymatic complex. It is important to highlight that all mutations associated with familial type of AD (APP, PS1, and PS2), in one way or another, increase A*β* production or modify its production rate [26] (Figure 1).

## 2. Toxic Mechanisms of A*β*

**Figure 2:**
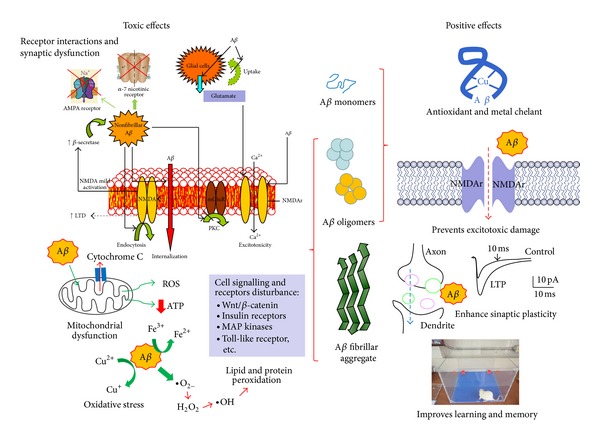
Illustration that summarizes the main negative and positive effects that have been experimentally described for A*β*. (a) Toxic effects (left): synaptic dysfunction, excitotoxicity, mitochondrial dysfunction, oxidative stress, and alteration of cell signaling pathways. (b) Positive effects (right): antioxidant, metal chelator, increasing synaptic plasticity, preventing excitotoxicity, and stimulating learning and memory.

It has been experimentally demonstrated that A*β* owns different and antagonistic biological characteristics: on one side, it has trophic and even antioxidant properties; on the other side, it has a high diversity of toxic mechanisms [7]. Multiple studies have revealed that A*β* toxic properties are mediated by several mechanisms, like oxidative stress, mitochondrial diffusion, alterations in membrane permeability, inflammation, synaptic dysfunction, excitotoxicity through its interaction with some neurotransmitters receptors, and so forth [27–31]. Next, we will further comment on the main toxic mechanisms that have been demonstrated for the A*β* (Figure 2).

### 2.1. Oxidative Stress

The pro-oxidant effect shown by the A*β* has been broadly examined using the paramagnetic electronic resonance (PER; a highly-sensitive method for direct detection of free radicals) [32]. High doses of this peptide are usually required so as to observe this effect, which is further improved when a peptide on either aggregated or fibrillar state is used [33]. Nonetheless, the precise mechanism by which such an oxidative effect occurs is still a matter of debate. Whole A*β* has some metal-binding sites in its first 15 amino acids constituted by the histidines 6, 13, and 14 and the tyrosine in the 10 position, all of which have well-known and powerful metal-binding sites, particularly for Cu^2+^, and a nearby affinity to the best metallic chelants currently known [33]. It has also been revealed that Cu^2+^ can be bound by the nitrogen atoms contained in the histidines' imidazole rings and it is suggested that the necessary oxygen to enable this binding can be provided from either the hydroxyl group of the tyrosine 10, the carboxylated lateral chain of Glu_5_, the ending amino group, or a water molecule [34]. Recently, a binding site for the Cu^1+^ at the 13 and 14 histidines has been located, though this site has yet not been implicated in any redox reaction [35].

The A*β* possesses the ability to reduce Cu^2+^ and Fe^3+^ towards Cu^+^ and Fe^2+^, respectively. This way, the molecular oxygen can react with reduced metals thus generating superoxide anion, which in turn combines with two hydrogen atoms to form hydrogen peroxide that may later react with another reduced metallic ion and then forming the hydroxyl radical by Fenton reaction. The A*β*—in its radical form—can extract protons from the neighboring lipids or proteins, thus generating lipid peroxides and carbonyls, respectively [34]. The role of metals in the A*β*'s toxicity has been fully demonstrated by experiments in which, either by their entire withdrawal from the reacting medium or by using deferoxamine, significantly lowered toxicity levels showed by A*β* in cellular cultures [35, 36]. It is else wise thought that these metals' reduction is mediated by a 35 position-located methionine, whose sulfide group has the ability to oxide and, therefore, easily donate electrons. In this sense, several studies have revealed that when this amino acid is substituted, A*β*'s oxidative properties are completely removed [37]. Supporting this hypothesis, copper-bound methionine sulfoxide has been found within the amyloid plaques of AD patients [38]. However, one study recently confirmed the oxidation of neurotransmitters when exposed to A*β*'s 1–16 and 1–12 peptides bound to metal but lacking Met35 residue, thus keeping this residue's role under discussion [39]. In parallel with the hypothesis of Met35 as a source of the electron involved in the metals reduction, the existence of an external reducer, like dopamine or ascorbate, is proposed as well, since this may permit the beginning of metallic ions' redox cycles without needing peptide autoxidation. Furthermore, formation of tyrosyl radicals from 10 tyrosine of the A*β* is involved in the cross-bridge dityrosine link in between two molecules of A*β*, therefore contributing to the formation of A*β* oligomers [30, 34]. As an additional mechanism, an increased expression of the divalent metal transporter 1 (DMT1) in the senile plaques of AD patients has recently been demonstrated, in APP/SS1 transgenic mice, and even in cellular lines overexpressing APP, all of which have been correlated with increased levels of iron in human cells exposed to A*β* and also in the *C. elegans* that also overexpress APP. It is therefore suggested that such impairments in iron homeostasis could contribute to an increase in oxidative stress caused by A*β* [40].

### 2.2. Synaptic Dysfunction

Many studies have shown that severity of synaptic loss is better correlated with cognitive impairment of AD patients rather than with the number of A*β* deposits or neurofibrillary tangles [41]. Some studies demonstrated a decrease of 25–30% in the number of cortical synapses in the AD, plus a 15–35% decrease in the number of each neuron's synapses. Some other studies have reported a protein reduction both presynaptic (synaptophysin) and postsynaptic (synaptopodin and PSD-95) in patients with AD in respect to healthy controls [42]. Also, it has been confirmed that disturbances of synaptic transmission occur long before the development of typical neuropathological lesions in the transgenic models of AD [43]. *In vivo* and in tissue-slices studies have shown that A*β* soluble oligomers, both synthetic and naturally secreted, can reduce the long-term potentiation process (LTP), yet this result was not replicated by the fibrillar forms of A*β* [44]. The mechanism by which A*β* oligomers disturb the synaptic transmission is unknown, but it has been reported that A*β* decreases the levels of PSD-95 and is further able to negatively regulate the AMPA (GluR1) and NMDA glutamatergic receptors through several mechanisms, like endocytosis, a reduction in the expression of some subunits, and so forth [45]. Although a lot of attention is currently focused on the damages produced by soluble forms of A*β*, some experimental studies have also demonstrated that fibrillar forms at least contribute with persistence to synaptic damage in the AD [46]. Nonetheless, some authors do suggest that synaptic injury that develops all along the course of the disease is also produced by soluble forms of the A*β*, based on the theoretical constraint stating that fibrillar forms already deposited may have a lesser probability of dynamically interacting with the receptors or proteins located at the synaptic terminals [45]. By using A*β* aggregates, some studies have demonstrated them as capable of inhibiting the NMDAr-dependent LTP, with no modulation over the NMDAr-independent LTP. Furthermore, it has been revealed that oligomers from different origins can promote some other plastic synaptic processes, such as long-term depression (LTD) in the CA1 neurons of the hippocampus, and this result was then associated with an increase of extracellular glutamate, which suggests this may be a consequence of an alteration in the glutamate reuptake [47]. Although several studies have unveiled the A*β*'s damaging effect over synaptic transmission, in fact little was known regarding it may depend on a pre- or postsynaptic mechanism. Having this in mind, two recent and parallel experiments examined the outcome from both extra- and intracellular administering of A*β* oligomers, and they found that intra-axonal administration of A*β* 1–42 (but not A*β* 1–40) in an experimental preparation of a giant squid's axon did alter the synaptic transmission measured by electrophysiological parameters, as well as the bidirectional fast axonal transport by a pathway depending on the activation of the casein kinase 2 (CK-2). It is worth noting that both studies did not demonstrate any effect with the administration of the A*β* oligomers at the extracellular level [48, 49]. On the other hand, one experimental report revealed that the synaptic action not only lowered the intracellular levels of A*β*, which may be partly due to the effect of neprilysin, but also promoted its extracellular secretion, which is associated with a decrease of the synaptic toxicity. Thus, this evidence strongly supports the hypothesis that A*β*'s main toxicity mechanism is intracellular. Recently, it has further been shown that within the A*β*'s negative influence over the synaptic functionality, Tau protein has a very significant role, given the fact that it was demonstrated that hippocampal slices from Tau-protein knocked-out animals are resistant to the harmful effects of A*β* 1–42 over the LTP [50].

### 2.3. Interaction with Receptors

#### 2.3.1. NMDAr

A*β* interaction with various receptors, channels, and other membrane proteins is well demonstrated [51]. For instance, A*β* interaction with different neurotransmitters receptors is considered as one of the most transcendental pathological events in the origin of AD, both in the synaptic dysfunction associated with cognitive impairment as well as in the processes leading to direct neuronal injury (i.e., excitotoxicity) [52]. The almost immediate (and sometimes transient) deleterious effects of the acute administration of soluble forms of A*β* on synaptic plasticity paradigms (LTP or LTD) clearly stated that such effects are mediated by mechanisms that are not dependent on neuronal toxicity or neuronal death, but on rapid effects on the synaptic neurotransmission. On the other hand, it has been revealed that these effects can be prevented by using an NMDA receptor antagonist, which suggests they may be mediated by A*β* action over the NMDAr. In addition, this processing is apparently associated with calcineurin and cofilin activation, the latter being a cofactor of actin depolymerization, which is contained in the dendritic spines. Such mechanisms could improve the LTD and inhibit the LTP [41]. However, the actions of A*β* on the NMDAr are complex and contradictory. The first results on the interaction of A*β* with the NMDAr arose from experiments that tested NMDA antagonists for protection or reversion of damage caused by A*β*, like the MK-801 [52–55]. Nonetheless, the relationship between A*β* and NMDAr seems to be more complicated. In some other experiments, mild stimulation of the NMDAr incremented the synthesis of A*β* by promoting a change in the predominant activity of *α*-secretases towards *β*-secretases [56]. In this sense, a recent study demonstrated that only the activation of the extrasynaptic-located NMDAr increased the A*β* production, whereas the activation of synaptic NMDAr did not evoke the amyloidogenesis [57]. Moreover, some additional studies have reported that NMDAr functionality is required for internalization and accumulation of the A*β* 1–42, since the use of NMDA antagonists reduced the cellular internalization of A*β* [58]. On the other hand, the use of A*β* 25–35 and 1–42 can decrease the number of NMDA receptors in the neurons [59], either by decreasing the expression of several subunits or by promoting their endocytosis [60]. Besides these results on the NMDAr, one study suggests that A*β* can also reduce the glutamate reuptake (by inactivation of the EAAC1 transporter), thus increasing the extracellular concentration of glutamate and promoting a receptor desensitization, which would improve the LTD and inhibit the LTP [47]. Although the way in which NMDAr is affected by A*β* has been studied deeply, little is known about how this interaction actually happens. Some studies have demonstrated a colocalization of the A*β* and the NMDAr, thus supporting the idea about its straight communication through glycine and glutamate sites. However, some other works have suggested that receptor modulation depended on the A*β*'s interactions with other molecules, like voltage-sensitive calcium channels, nicotinic receptors, insulin receptors, or metabotropic receptors, all of which could modulate NMDAr activity in a secondary way [47]. In this respect, Texidó et al. [61] used *Xenopus* oocytes that expressed NMDA channels (NR2A and NR2B) and showed that when A*β* soluble oligomers 1–42 (100 *μ*M) were added, input currents evoked by these channels were reversed with various NMDA antagonists (APV, MK-801, and memantine); this was moreover correlated with an increment in the cytosolic calcium. This same study also found that currents evoked by the direct administration of A*β* were larger for the NR2A receptors, which suggest an A*β*'s higher performance over this receptor's subpopulation [61]. Previously described evidence strongly accounts for a direct interaction of the A*β* with certain NMDAr subpopulations, although neither the precise site of this A*β*'s interplay with the NMDAr nor the peptide's residues that may have a key role for such an interaction are well understood yet. Furthermore, an additional study made in hippocampus slices revealed that the application of a NR2B-specific antagonist together with a negative allosteric modulator of the mGluR5 metabotropic receptors can reduce the negative effects of the oligomers of A*β* 1–42 over the LTP, these results being comparable with those obtained when using memantine and, therefore, suggesting that these glutamatergic receptors could have an important participation in the damage caused by the A*β*, which underscores them as possible pharmacologic therapeutic targets for the future [62].

#### 2.3.2. AMPAr

It has been reported that A*β* exerts both a decreasing in AMPA receptors (AMPAr) activation as well as a reduction in the amount of the same receptors at the postsynaptic level. The mechanisms by which these changes may happen are believed to be the following: (a) the A*β* increases the activity of Caspase 3, which has shown a proteolytic activity over the AMPAr; (b) the A*β* inhibits the auto-phosphorylation of the CA2+/Calmodulin II-dependent protein kinase (CaMKII), which in turn phosphorylates the AMPAr; (c) the A*β* induces a direct phosphorylation of the GluR2 subunit thus promoting the endocytosis of the receptor; (d) the A*β* produces the endocytosis of the AMPAr by means of activating the signaling involved with the LTD mediated by p38, MAPK and calcineurin; (e) another possibility is that A*β* stimulates the extrasynaptically-located NMDAr, which may be coupled with the p38 MAPK pathway, and (f) it exists the possibility that signaling pathways activated through inflammation produced by A*β* would also converge in the activation of p38 MAPK [63]. A recent study has confirmed such A*β*'s adverse effects over AMPAr, by finding that, in the hippocampus neurons, the oligomers of A*β* 1–42 reduced the number of AMPA postsynaptic receptors, further diminishing the membrane insertion of new AMPAr as well as the mitochondrial transport and translocation towards the dendritic spines [64]. In contrast, other studies have suggested that A*β* has an activating effect over AMPAr, by proving that engaging of this receptor is at least equally important for the neuronal injuries provoked by the administration of oligomers of A*β* 1–42 to both neuronal cultures and entorhinal-hippocampal organotypic slices [65].

#### 2.3.3. Cholinergic Receptors

It is well known that A*β* 1–42 binds with a significantly greater affinity to the *α*7-nicotinic receptors than 1–40 and it is proposed this binding has a significant participation on the internalization and accumulation of A*β* in cholinergic neurons, a point that has been demonstrated when successfully blocking the internalization and accumulation of A*β* 1–42 using antagonists of *α*7 receptors [66]. Since A*β* use to predominantly accumulate in neurons that have *α*7-nicotinic receptors, it has been suggested that sole presence of this receptor may be an underlying factor for the selective cellular toxicity shown by A*β* in the brain of AD patients [67]. Using *α*7, *α*4*β*2, and the recombinant *α*3*β*4 nicotinic receptors, it was revealed that A*β* 25–35 reduces the currents mediated by the three receptors, whereas the A*β* 1–42 reduces the currents mediated by *α*7 only, increases those mediated by *α*4*β*2, and does not affect those of *α*3*β*4 [68]. On the other side, there are several reports in which a dose-dependent decrease in the total number of muscarinic receptors after *in vivo *exposition to A*β* 25–35 was found [69]; the same results have been found in transgenic mice that overexpressed A*β* and whose M2 receptors were reduced at the cortical level [70]. In contrast, there is evidence supporting a protective role of the *α*7-nicotinic receptors at the initial physiopathologic stages of AD. One of such studies used transgenic mice for PPA (Tg2576), which were also knocked-out for the *α*7 receptor, and found a more severe cognitive impairment as well as an increment in the A*β* oligomers accumulation when compared with the Tg2576 mice that indeed expressed the receptor. It is thus suggested that *α*7 receptors could have a protective role, at least in the disease's beginning [71].

### 2.4. Mitochondrial Dysfunction

Recently, the intracellular accumulation of A*β* was associated with the neuronal damage seen in the AD [72]. The A*β* has been found in membranous intracellular structures like the endoplasmic reticulum, the Golgi system, lysosomes, endosomes, and in the mitochondria's inner membrane or matrix [73]. Nevertheless, the origin of mitochondrial A*β* is uncertain. It has been known for some time that APP is usually located in the mitochondrial external membrane, but to date, enzymes with beta-secretase activity have not been found at the mitochondrial inner membrane, being actually the gamma-secretase class the only the only enzymatic activity found there [42]. This suggests two alternatives: either that products of beta-secretases are transported to the mitochondria (from other cellular sources), where their proteolysis by gamma-secretases end, or that A*β* peptide is completely elaborated on a separate site and then moved inside the mitochondria [73]. The transport towards the mitochondrial inner entails another problem and for now the transporting system that A*β* may use is not completely understood [73]. Some authors have suggested that A*β* could bind to and be transported by some proteins, like the alcohol dehydrogenase [74]. In fact, it was recently reported that A*β* can be moved inside the mitochondria using the mitochondrial outer-membrane translocase (TOM); moreover, the authors suggest that A*β* is predominantly located in the inner membrane's cristae [75]. It also exists a tight temporal and sequential relationship between the collection of mitochondrial A*β* and its own dysfunction [73]. *In vitro* studies have shown that exposition of mitochondria to A*β* induces a decrease in the respiratory states 3 and 4, as well as a decline in the activity of cytochrome c oxidase and some other Krebs cycle's enzymes [76]. Other studies have revealed that, in the presence of calcium, A*β* can create transition pores into the mitochondrial membrane through which cytochrome C can be released and, therefore, proapoptotic signaling pathways can be started [77]. A recent study even demonstrated that A*β* can directly inhibit the generation of mitochondrial ATP, then affect the correct functioning of alpha-subunit of ATP synthase [78]. According to some other reports and besides altering the enzymatic mitochondrial machinery, A*β* administration in subtoxic doses and in a chronic manner can inhibit the transportation of nuclear proteins to the mitochondria, which is further associated with impairment in its membrane potential and the production of reactive oxygen species (ROS) [79]. Activation of enzymes like the NADPH oxidase, the xanthine oxidase, and the A2 phospholipases (in both cytosolic and calcium-dependent forms) have also been involved in the mitochondrial dysfunction and production of ROS by A*β*. When such enzymes are pharmacologically blocked, ROS production and mitochondrial dysfunction by A*β* are significantly reduced [73]. Finally, it has been seen that tissue samples from patients with AD have a larger number of mutations in the mitochondrial DNA, and also that expression of mitochondrial genes is augmented in transgenic mice overexpressing the APP, animals that also show higher levels of A*β*. This increment in the genes expression has been interpreted as a compensation mechanism against mitochondrial dysfunction caused by the A*β*, though it has been reported that various deletions of mitochondrial DNA can also occur in normal aging [80]. Finally, all these mitochondrial alterations derive in variations of the mitochondrial structure, which might underlie the fact that neurons exposition to A*β* breaks the typical mitochondrial cords organization [80]. All this evidence recently led to propose the *mitochondrial cascade hypothesis* of AD, which holds that inherited mitochondrial genes, mitochondrial dysfunction associated with aging, and deficiency in antioxidant mechanisms coupled with a low intake of exogenous antioxidants, promote a severe redox imbalance, thus resulting in an increased production of A*β*, which, if excessive, may boost the mitochondrial dysfunction initiating, therefore, a vicious cycle that eventually leads to neurodegeneration [81].

### 2.5. Interaction with Membranes

Fluorescence studies showed that A*β* strongly and quickly binds with all cellular membranes [82]. In addition, it is well established that cellular exposure to A*β* generates an increment in the intracellular calcium, which is closely related with several processes of damage and cell death. However, the mechanism by which this increment in the intracellular calcium is evoked is not well understood. A great variety of A*β*-activated receptors and channels have been involved, but it is also known that A*β* can directly interplay with the lipid components of the cell membrane, forming pores or ionic channels that help with Ca2+ entering into the cell [82, 83]. There are many studies proving the insertion of the A*β* 25–35 into the lipidic membranes [84, 85], and even some researchers do agree that this fragment forms nonspecific channels in a faster and more efficient way than full peptides do [86]. On the other hand, it is well established that composition of cell membranes can influence the general toxicity shown by the A*β* by affecting its aggregation, though this seems to be valid only for the A*β* 1–42, since A*β* 25–35 appears not to be affected in its aggregation due to the composition of the membrane lipids [85]. As expected, metals added during the interaction of membrane and A*β* 25–35 produced no further effect, which instead did happen with the whole peptide, therefore increasing A*β* aggregation and toxicity [87]. There is evidence showing that high levels of cholesterol increment A*β* synthesis and also facilitate its interaction with the membrane [88]. The presence of pores or ionic channels formed by A*β* has been proved by specific blockade of these channels, thus reducing both calcium entering and neuronal damage very significantly [82].

### 2.6. A*β* Signaling Pathways and Other Receptors

Besides direct multiple toxic effects exerted by the A*β*, it has demonstrated to promote activation of several signaling pathways inside the cell through its binding to many cell's receptors (insulin, RAGE, NMDA, nicotinic, etc.). These signaling pathways can notably participate on the neuronal damage observed in the AD, but they also represent a general mechanism through which distinct involved physiopathological factors (e.g., tau hypothesis, amyloid hypothesis, calcium role, etc.) lead to the AD. Likewise, these pathways are promising molecular therapeutic targets, with a high potential for the AD manage. Next, we will review only some of the signaling pathways in which A*β* has been implicated.

#### 2.6.1. *Wnt*/*β*-Catenin

The *Wnt* is a pathway recognized as essential in the embryonic development as well as by its known role in the oncogenesis. Furthermore, this pathway has been recently described to have an important role in the origin of some neurodegenerative disorders [89]. The *Wnt* pathway also has three principal branches for intracellular signaling: (1) the *β*-catenin pathway or canonic pathway, which in the end activates different target genes at the nuclear level; (2) the flat-cell polarity pathway, which involves the jun N-terminal kinases (JNK) and various modifications of the cytoskeleton, and (3) the Wnt/calcium pathway. The Wnt canonic pathway stabilizes the cytosolic *β*-catenin to have it entering into the cell nucleus and binding it with the LEF/TCF transcription factors, which in turn activate the transcription of many target genes (conductin D1 cyclin, En-2, ID2, MMP7, Myc, PPAR*δ*, etc.). In the absence of Wnt signaling, *β*-catenin is phosphorylated by CKI*α* and/or CKI*ε*, as well as by GSK3*β*, all of which trigger its ubiquitination and subsequent degradation through a proteasome [90]. Some studies have shown that PS1 can form high-molecular-weight complexes with the *β*-catenin protein and also with the GSK3*β*, leading to suggest that PS1 could act as a regulator/coupler at the interaction between tau and GSK3*β*; it also has been reported that patients with mutations in the PS1 have decreased levels of *β*-catenin together with a change of this protein translocation towards the nucleus, which suggests an impairment in the Wnt pathway. Besides the synthesis enhancement of AB, these findings suggest that PS1 mutations observed in early-onset AD also modify the signaling of Wnt pathway, turning it into an additional pathological mechanism. [91]. Moreover, there is also evidence that the toxicity exerted by soluble A*β* can affect the Wnt pathway both indirectly (altering its intracellular signaling), as well as directly binding to the extracellular domains of the frizzed receptor protein inhibiting the Wnt/*β*-catenin pathway [92, 93]. On the other hand, there is extensive therapeutic data about the use of lithium (a reversible inhibitor of GSK3*β*), proving it prevents the neuronal damage caused by the A*β* as well as the tau hyperphosphorylation and the subsequent loss of the neuronal microtubular network [94]. Similarly, activation of the Wnt pathway using the *Wnt-*3a endogenous ligand prevents harming induced by A*β*, this effect being reverted by using a sFRP pathway antagonist [95]. Moreover, GSK3*β* inactivation mediated by the PKC (which inhibits GSK3*β* through 9-serine phosphorylation) reproduces such protective effects [96]. Beyond its effects over the tau protein, the canonic signaling of the Wnt pathway has been involved in the development of dendritic arborization, and it also modules the expression of some synaptic proteins like the synaptophysin and the PSD95. All these evidences not only suggest that a decrement in the canonic signaling of the Wnt pathway not only plays a role in the neuronal damage observed in the AD, but also that this pathway could be the joining point of the pathologic processes generated by the A*β* and the tau protein, and thus a very significant target for AD treatment [97]. In fact, a therapeutic effect has recently been reported of various compounds that act upon this pathway; such is the case of the muscarinic agonists that activate the Wnt pathway through the PKC activity, so inhibiting the GSK3*β*. Furthermore, in some *in vitro* studies, a few compounds like curcumin can activate the Wnt pathway by inhibition of the GSK3*β* expression [98]. It is also worth remarking on the essential involvement of the Wnt/*β*-catenin pathway in the neurogenesis process that occurs in the adult brain [99].

#### 2.6.2. Insulin Receptors

Many studies both clinical and epidemiological have unveiled a strong association between peripheral resistance to insulin and the risk to develop AD [100]. The impairment in the insulin receptors (IR) within the AD is well known, which can lead to expression decreasing, desensitization, and even alterations on their intracellular signaling pathways. The IR signaling initiated by a ligand activates the autophosphorylation of tyrosine residuals in the *β*-subunit, which stimulates two intracellular pathways: the phosphatidylinositol 3-kinase (PI3K)/Akt and the mitogen-activated Ras/protein kinase (MAPK) pathways. The PI3K/Akt pathway is vital for metabolism and neuronal survival, and it is known that its inactivation elevates the neuronal death rate due to oxidative stress and excitotoxicity [101]. *In vitro* studies have shown that incubation of hippocampal neurons with 1–42 A*β* oligomers (100nM) for 30 minutes is enough to provoke an IR diminution at the dendritic level and an increment of these receptors in the neuronal soma. At the same time, impairment in the IR signaling when responding to stimulation was also observed, which was associated with the phosphorylation of Akt in the 473 serine; such a modification is considered as inhibitory for the IR signaling [102].

#### 2.6.3. MAPK

The family of protein kinases activated by mitogen (MAPK) includes kinases regulated by extracellular signals or ERK; the c-Jun N-terminal kinases, or JNKs, and the p38 protein. JNK and p38 pathways are particularly activated by many stressing stimuli (e.g., oxidants, tumor necrosis factor, ultraviolet radiations, etc.), so that their activation is generally associated with the induction of cell apoptosis. On the other hand, the ERK activation conveys an important role in the neuronal growth and differentiation processes [34]. For instance, it is well known that ERK2 signaling cascade has a transcendental function at the hippocampal synaptic plasticity that takes place during learning. In the hippocampus' CA1 region, the ERK2-MAPK cascade is necessary for expression of the late-phase of the LTP, and it is a very important pathway by which neurotransmitters mediate the LTP induction. In addition, it has been revealed that this signaling pathway has a key role for the correct performance at particular tasks of associative learning [103]. A study based on organotypic hippocampal slices showed that the addition of 1–42 A*β* (100 nM) incremented the ERK2-MAPK pathway's activation, and also that such a modulation was mediated by the A*β* effect over nicotinic *α*7 receptors. Together, these results suggest that the chronic activation of A*β*-mediated ERK2-MAPK cascade may eventually downregulate itself, thus affecting CREB phosphorylation, the expression of nicotinic receptors and, finally, generating a disturbance in the LTP [104].

#### 2.6.4. Toll-Like Receptors

A study performed in neuronal cultures showed an increment in the expression of toll-like receptors (TLR4) when exposed to 1–42 A*β* at concentrations that started from 1 to 10 *μ*M, which was linked with the appearance of JNK-mediated neuronal apoptosis. It was likewise proved that neurons of a TLR4-knocked-out mouse showed resistance to apoptosis induced by 1–42 A*β* [105].

### 2.7. A*β* and Reentry in the Cellular Cycle

It was classically considered that all mature neurons were stationed at the G0 stage. However, recent reports have shown that, in some pathological states such AD, Parkinson's disease, amyotrophic lateral sclerosis, and so forth, several neurons can exhibit molecular markers that suggest a reactivation of the cell cycle, and even some of them can also finish their synthesis of genetic material, therefore completing the S phase of the cell cycle [106]. In the AD, this reentering to the aberrant cell cycle precedes to the cell neurodegeneration process, thus representing an early cell indicator of neuron's susceptibility to cell death [107, 108]. Recently, a study based on primary cultures of cortical neurons found that 24-hours incubation with 1–42 A*β* oligomers elicited markers of reentering in the cell cycle, which did not occur with the incubation of either monomeric or fibrillar A*β*. It was simultaneously shown that neurons treated with A*β* highly expressed Akt and mTOR (mammalian Target Of Rapamycin), and when inhibitors for this signaling pathway were used, A*β* effects over the cell cycle were blocked, suggesting this pathway could be early affected by the oligomeric A*β*, perhaps through its interaction with IR [109].

## 3. Positive Effects of the A*β*

The protective effects of A*β* have been little studied, ignored, or left aside. The vast majority of published scientific articles on this respect consider A*β* as a fundamental component of the toxic mechanisms seen in the AD. Nevertheless, up to date there are significant amounts of scientific data that have demonstrated that, under certain conditions, A*β* can show some protective, trophic, or even antioxidative physiologic effects. In a first sight, both pathological and physiological A*β* effects seem to be contradictory, but in reality the available evidence suggests that such properties could not be mutually exclusive, since conditions in which toxicity occurs used to be completely different from those occurring during protective effects. For this reason, many authors have suggested that, in fact, basal production of A*β* merely entails a physiologic role in the nervous system and that, under certain circumstances, its production, clearance, or physical state might be altered, thus turning this physiologic function into a toxic, pathologic effect. Next, we will review what we consider the most important evidences about protective, physiologic mechanisms experimentally revealed by the A*β* (Figure 2).

### 3.1. A*β* as an Antioxidant

The A*β* is a peptide of 39 to 42 amino acids that in its inner structure has two essential sites for its redox function. The first site is localized in the peptide's N-terminal hydrophilic part and is constituted by histidines 6, 13, 14, and a 10-positioned tyrosine. This site has the particular property of binding transition metals efficiently, thus lowering the possibility these metals may get involved in some other redox reactions that could increase the oxidative damage. The second site is located in the peptide's C-terminal lipophylic portion and is constituted by just one methionine residue at the 35 position. This residue has two opposed properties: in one side, it can trap free radicals, but in the other, it can reduce metals and then turn them into more reactive forms with a lower valence, therefore having both anti- and pro-oxidative effects [110]. Also, it has been proven that A*β*'s metal-binding ability is better for copper (Cu) than for iron (Fe) and its affinity quite equals that shown by the best known chelants, like the ethylenediaminetetraacetic acid (EDTA). In comparison with its constant and uniform ability to chelate metals, the A*β*-mediated reduction of transition metals happens very slowly, which suggests that its predominant role in standard circumstances may correspond to that of an endogenous *scavenger.* In addition, it is known that 1–42 A*β* is a more potent chelant than 1–40 A*β*, which is well correlated with the former's higher-reducer characteristics.

Several cell studies have confirmed these protective, antioxidative effects of the A*β*. In nanomolar concentrations, the A*β* can reduce the apoptotic death in neuronal cultures once the administration of trophic factors is suspended [111], and it is further suggested that this antiapoptotic result is closely related with the A*β*'s chelating ability over some metals, Cu in particular. Some other *in vitro* studies have shown that A*β* monomers decrease the oxidation of lipoproteins in the cerebrospinal fluid (CSF) and blood plasma [33, 112]. Furthermore, CSF's resistance to oxidation is better correlated with A*β* levels rather than with ascorbate levels, which is considered as the CSF's most important antioxidant property [113]. As expected, this CSF's antioxidative aspect is also better correlated with levels of 1–42 A*β* than with levels of 1–40 A*β*, given the former's superior role as a metal chelant [7]. Cells that overexpress A*β* seem to have a decreased production of ROS and a lower susceptibility to be damaged by metals. In cultures of cortical neurons, either incubation with inhibitors of *β*- and *γ*-secretases or aggregation of antibodies against A*β* significantly reduced cell viability; notably, this effect was completely reversed by adding 1–40 A*β* [114]. In a related study using cultured neural stem cells (NSC), it was shown that oligomers of 1–42 A*β*, in concentrations of 1 *μ*M, significantly increased survival and differentiation of striatal and hippocampal NSC; again, this effect was neither seen when adding 1–40 A*β* or 25–35 A*β*, nor with fibrillar forms of these peptides [115].

Initial *in vivo* studies demonstrated that hippocampal implants of A*β* in 3- and 18-month-old rats did not provoke any neurotoxic effect from the morphological point of view [116]. Subsequent studies with chronic administration of various A*β* peptides (1–40, 1–38, 25–35) at different dosages (5 ng–10 *μ*g), applied in the cortex and hippocampus of adult rats, did not produce any particular toxic effect compared with control [117]. The intracerebral administration of low-concentrated A*β* in young animals (monkeys and rodents) did not result in neuronal damage, whereas it did affect neurons in older animals. Reasons for these differentiated results depending on subject's age are not well understood, but it is speculated that it could be due either to the high content of free metals in the brain of older animals or to the reduction of the antioxidative defenses that occur with age [118]. The possible role of A*β* as an antioxidant is also supported by the fact that in models of mitochondrial dysfunction using inhibitors of complex I and III (rotenone and antimycin), an increment in the oxidative stress occurs in association with a significant increase in the A*β* production and interestingly, this increase is reversed by the use of antioxidants [119]. All previous evidence about the antioxidant effects of A*β* have been demonstrated using nonfibrillar forms; however, a recent study seems to show that even in the aggregate state and in concentrations of 2–20 *μ*M the A*β* is able to reduce the formation of hydroxyl radical and hydrogen peroxide in synthetic nonbiological systems and may be further able to prevent oxidation of proteins and lipids in isolated mitochondrias from rat brain [120].

All this evidence led some authors to suggest that a main physiological task of A*β* may be to act as an endogenous antioxidant, which would explain the fact that in normal aging (where oxidative stress is increased) the production of A*β* is also augmented. In this context, the AD would then produce a chronic and severe redox imbalance state that the overproduction of A*β* eventually could not compensate anymore, thus becoming toxic. In this respect, some authors argue that the A*β* should not be seen as the initiator of the pathological process, but as the consequence of an underlying oxidative pathological process [121].

### 3.2. A*β* as a Neuroprotector

Giuffrida et al. observed in neuronal cultures that administration of synthetic A*β* 1–42 monomers in concentrations of 0.1 *μ*M prevented the cell death induced by deprivation of trophic factors, like insulin, and in concentrations of 30–100 nM protected from excitotoxic effects of NMDA, when administered both before and after the excitotoxic stimulus. Similarly, it was demonstrated that during this protective effect the phosphatidylinositol 3-kinase (PI-3K) pathway was activated. Interestingly, when A*β* 1–42 monomers with the Arctic (E22G) mutation were used, no neuroprotective effects were observed, possibly because this mutation alters very significantly the peptide conformation, thus affecting their protective properties [122]. A similar study has confirmed that the nonfibrillar A*β* 1–42, in concentrations up to 1 *μ*M, was able to reduce the cell death and intracellular calcium entry induced by NMDA receptor activation, but it failed to produce a protective effect with AMPA receptor activation [123].

### 3.3. Electrophysiological Studies

Initial electrophysiological studies of hippocampal slices showed that A*β*, in nanomolar concentrations (100–200 nM), facilitated the LTP and increased the synaptic currents of the NMDAr, without affecting the AMPAr currents [124, 125]. A subsequent study carried out in hippocampal slices demonstrated that administration of A*β* 1–40 (83 nM) restored the ability to generate LTP previously affected by prolonged incubation of the slices and also showed that inhibition of the synthesis of cholesterol reversed this effect. The authors, therefore, suggested that the A*β* 1–40 facilitates the dynamics and availability of membrane cholesterol [126]. A recent study has confirmed these findings, proving that very low concentrations of both monomers and oligomers of 1–42 A*β* (200 pM) applied to hippocampal slices enhance the LTP, and such result was behaviorally correlated with an increment of the reference memory and the context fear. This same study also showed that administration of *α*7-nicotinic antagonists suppressed the LTP, which suggests that the positive effect of A*β* over the synaptic plasticity may be mediated, at least partially, by the effect upon *α*7 receptors [127]. A subsequent and similar study using hippocampal slices reported that reducing the expression of APP by interference RNA also caused LTP reduction, which was further correlated with a decrement of spatial and contextual fear memories at the *in vivo* model. Interestingly, such effects were reversed by the exogenous addition of 1–42 A*β* of human origin [128].

Furthermore, it was recently shown in an *in-vivo* study in rats that the sequestration of endogenous A*β* (using hippocampal infusion of a monoclonal antibody against the ectodomain of the A*β*), performed immediately before training, significantly altered the retention of short- and long term-memory in an inhibitory avoidance task, while this parameter was not affected when the antibody was administered after training session. These results were identical to those obtained by administering a nicotinic cholinergic receptor antagonist (mecamylamine). Interestingly, this same study also showed that the negative effect on learning was reverted by exogenous hippocampal administration of human A*β* 1–42 (100 pM), further demonstrating that the A*β* 1–42 also promotes memory consolidation when administered after training [129]. In a similar study conducted *in vitro* and *in vivo*, the authors demonstrated that concomitant administration of anti-A*β* antibodies and interference RNA modified both the LTP as spatial reference memory and the contextual fear conditioning, and that these parameters were recovered by the administration of A*β* 1–42 at concentrations of 200–300 pM. Interestingly, the authors also found that positive effects of A*β* 1–42 were absent in *knock-out* mice for the *α*7-nicotinic cholinergic receptor [128]. To further support previous evidence, the same authors recently conducted a dose-response curve to investigate the hormetic effect of A*β* 1–42 (2 pM to 20 *μ*M) over LTP and spatial memory in the Morris maze, finding that the stimulatory effects on the LTP of A*β* 1–42 was observed at doses between 2 pM to 2 nM, whereas for concentrations ranging from 2 nM to the 20 *μ*M negative effects on LTP were observed. Moreover, with a concentration between 2pm and 2 nM a reduction in escape latency was observed (i.e., it enhanced memory effect), whereas for a concentration of 20 nM an increase in escape latency was measured (i.e., it impaired memory effect) [130]. This latest evidence eloquently shows the ambivalent and dose-dependent effect that has been continuously reported in experiments carried out with A*β* on both synaptic plasticity and at the behavioral level when studying different types of hippocampal-dependent memory and further suggests that positive effects of A*β* may be associated with its direct action on *α*7-cholinergic nicotinic receptors, which have previously been involved in the regulation of glutamatergic transmission.

## 4. Conclusions

Nowadays, the accumulated experimental evidence leans toward strongly supporting the toxic role of A*β* within the pathophysiology of AD. However, the existence of some data regarding the A*β*'s role in the normal physiology of the brain does suggest that this peptide may act in different modes according to diverse conditions at different times. So far, it appears that at the initial stages of development and in the young brain, when in physiological doses (i.e., picomolar to nanomolar range) and in soluble, oligomeric forms, the A*β* can show neuroprotective, antioxidant, and trophic properties, even facilitating synaptic plasticity. On the contrary, in many potentially adverse conditions, the A*β* could deploy its multiple toxic effects thus contributing significantly to the neuronal damage, as seen in the AD. Some of these conditions appear to be associated with A*β* itself, like its high concentrations and fibrillar or aggregated states; presence of free metals; brain tissue previously injured or aged; and decreased antioxidative mechanisms. Moreover, it is necessary to remark that both trophic and toxic effects may not necessarily be mutually exclusive. In other words, they might be persistently coexisting and cross-modulating each other, even throughout advanced stages of AD, thus causing the approach based upon antiamyloidogenic therapy to be more complicated, at least theoretically. Moreover, this functional duality may also underlie the modest success and also the high rate of collateral consequences of such kinds of therapies. In summary, blockade, inhibition or modulation of those sites, effects and negative processes in which the A*β* is involved, but simultaneously respecting those sites and physiologic processes in which the A*β* is also taking part of, still remain as a major challenge for therapeutic research in the future.
